# Detection of Oil in Seawater Based on the Fluorometric Index during the Winter Season in the Baltic Sea—The Case of the Gulf of Gdansk

**DOI:** 10.3390/s22166014

**Published:** 2022-08-12

**Authors:** Emilia Baszanowska, Zbigniew Otremba

**Affiliations:** Department of Physics, Gdynia Maritime University, 81-225 Gdynia, Poland

**Keywords:** oil in seawater, oil detection, oil fluorescence, excitation-emission spectra of oil, fluorometric index, oil sensor

## Abstract

This study is a continuation of analyses of the fluorometric index (FI), based on the fluorescence of substances of oil origin, as an indicator of oil in a seawater column. The effectiveness of the FI in the cold season (late autumn, winter and early spring) for the coastal water in the southern Baltic Sea was assessed. FI was tested for seawater polluted with a mixture of crude oils, lubricating oils and fuels. Laboratory analyses of oil–water systems for low (reaching the limit of detection) oil content in seawater were performed. The influences of the natural components of seawater that disrupt oil detection are discussed. The ability to detect oil in a seawater column regardless of the season was confirmed.

## 1. Introduction

Various regulations have been introduced, such as The International Convention for the Prevention of Pollution from Ships (MARPOL) [1], along with regional and national legal regulations, to eliminate oil pollution in the marine environment [2]. These legal principles have led to a reduction in large oil spills. However, the potential risk of oil spills due to increased human activity cannot be completely eliminated. This is mainly due to the development of maritime transport and mining activities on platforms and the transport of hydrocarbons via pipelines [3,4,5]. Thus, pollution from shipping activities and subsequent ballast water discharges, such as crude oil, lubricating oil and fuel in small amounts, are still common [6,7,8,9,10]. Therefore, the continuous monitoring of oil pollution in endangered regions of the sea is advisable. It is therefore desirable to search for fast, simple methods of hydrocarbon detection and identification in seawater. 

The detection of oil contamination in seawater by various methods has seen extensive development. This has contributed to an increase in the efficiency of oil detection both on the sea surface and in the seawater column. Satellite or radar methods using airplanes and stacks are reliable and are commonly used to detect oil spills on the sea surface and over large areas, and they are still being improved [11,12]. Remote methods based on optical sensors using hyper-spectral remote sensing technology are also widely used [13,14]. However, the detection of an oil spill via remote methods has limitations due to weather conditions or the amount of light. Thus, the detection of oil at night using remote methods is a problem. When the oil is already in the seawater column, in situ methods are much better suited. In the field of oil detection in the sea, methods with various types of underwater sensors [15,16] are being intensively developed. However, what is needed is a low-cost and effective method of detecting oil in the place of the spill in the sea, in a short time. In this case, methods based on markers and probes to signal the presence of oil are increasingly being sought for the detection of oils [17].

Analyses were performed on the possibility of detecting a leak that was impossible to register with the use of equipment located above the seawater surface. Research was conducted on the possibility of detecting oil substances on the basis of changes in the seawater fluorescence spectra [18,19,20,21]. In these studies, difficulty was found to result from the partial overlapping of the fluorescence spectra from substances naturally occurring in seawater with the spectra originating from components of the oil. As a result of the analysis of the excitation-emission spectra (EEMs), a fluorometric index (FI) was defined, which indicates the possibility of seawater contamination with oil [22,23]. It was noted that the FI value is influenced not only by the relative amount of oil in seawater but also by the date of sampling of natural seawater, which is related to the seasonal changes in the composition and contents of natural seawater components. For this reason, it was decided to expand our research to seawater sampled in the cold season from November to March.

This paper provides an analysis of a proposed novel method for oil detection in seawater. The method is based on the FI which could be applied in underwater sensors as a potential indicator for oil detection in seawater. The analysis of the FI’s effectiveness in oil detection in seawater in relation to the cold season in the Baltic Sea was conducted in late autumn, winter and early spring, in the Gulf of Gdansk (Baltic Sea). The study is complementary to the authors’ previous paper [23], which involved laboratory tests performed in the warm season in the Baltic Sea basin.

## 2. Materials and Methods

### 2.1. Natural Seawater (Oil-Free) Samples

For the preparation of laboratory oil–water systems, samples of seawater from the coastal area were used [22,23]. Seawater was sampled from under the sea surface (at a depth of 1 m) in glass bottles [22,23]. Sampling took place in November, December, January, February and March in 2019/2020. In Table 1, the specifications of the main parameters of the seawater sampled are shown.

### 2.2. Contaminated Samples

**Figure 1:**
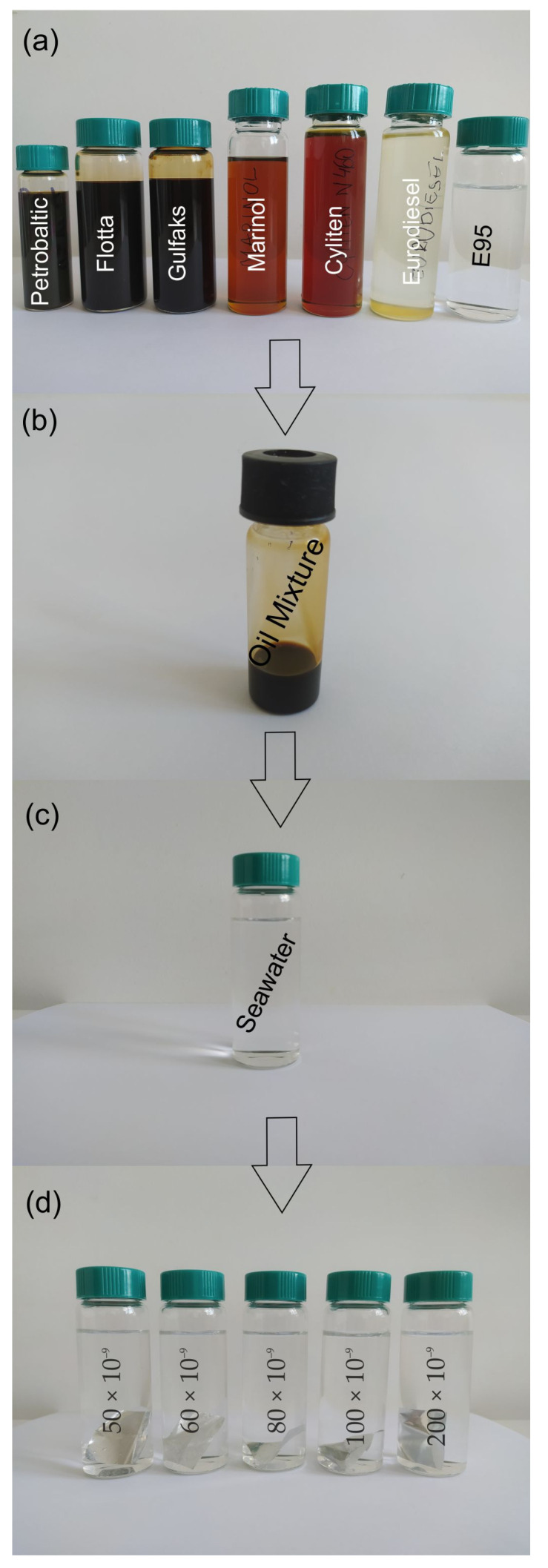
The steps of preparing seawater samples contaminated by a mixture of oils: (**a**) seven kinds of oils, (**b**) oil mixture, (**c**) seawater, (**d**) seawater samples contaminated by a mixture of oils.

Seawater samples were artificially polluted, as described by the authors in the previous paper [23]. The oil-free seawater samples were artificially contaminated by a mixture of oils consisting of crude oils, lubricating oils and fuels, which was previously weighted out on aluminum foil. The oil-free seawater samples were contaminated by the mixture of oils at several oil-to-water ratios (r_o/w_) in the range of 50–200 × 10^−9^ for each considered month in the cold season, from November to March. Finally, contaminated seawater samples were exposed to an additional mixture of oils for one day (illustrated in Figure 1).

### 2.3. Measurement and Apparatus

For the measurements of EEMs, the same tooling as in the paper [23] was used. Namely, a spectrofluorometer (Hitachi F-7000 FL) was used to measure EEMs of natural (oil-free) seawater samples and seawater artificially polluted by the mixture of oils. The measurements of EEMs were performed in a 1 × 1 cm quartz cuvette. The specifications of the measurement parameters are listed in Table 2. During the measurements, the temperature value in the measuring chamber of the spectrofluorometer was stabilized to room temperature: about 20 °C. To cool and stabilize the temperature in the measuring chamber of the spectrofluorometer, a Peltier circulation thermostat was used. The measurements of EEMs of seawater (oil-free) samples were performed three times in order to obtain the appropriate artefact-free background in relation to EEMs of the seawater samples polluted by the mixture of oils. Finally, to get a digital matrix of EEMs, Rayleigh scattering was removed.

## 3. Results 

### 3.1. Characteristics of Natural Seawater Samples

**Figure 2:**
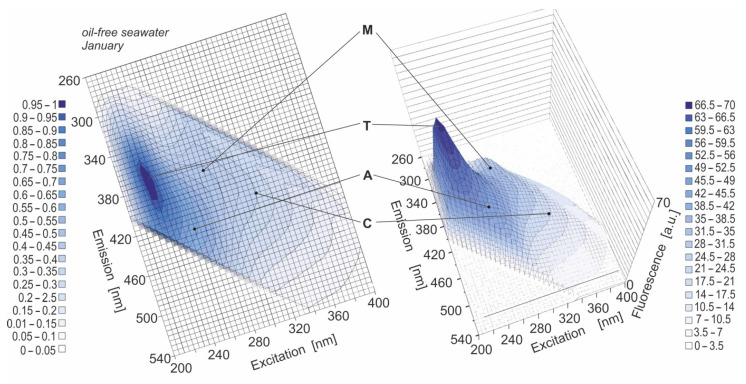
EEMs of oil-free seawater in 2D (**left** side) and 3D (**right** side) for the sample month of January in 2020.

Natural seawater samples have characteristic fluorescence spectra originating from the natural seawater components. Since the goal of the study was to obtain the information about the presence of oil in seawater polluted by oil in the cold season, and natural seawater components can affect the fluorescence of a mixture of oils, it was valid to determine the presence of natural seawater components which manifested themselves in the EEMs of natural seawater (oil-free seawater). Figure 2 presents the sample EEMs of natural seawater for the exemplary month—January. In the EEM spectrum, the specific peaks were determined and described by their wavelength-independent fluorescence maxima (λ_Ex_/λ_Em_): peak 1 (225/365), peak 2 (265/420), peak 3 (280/380) and peak 4 (320/415). The detected EEM peaks in Figure 2 were each marked by a black dot and denoted by a specific letter linked to a specific component of natural seawater based on the available literature data (see Table 3) [18,25,26,27]: a tryptophan-like seawater component (peak 1); humic-like A (peak 2); main humic-like M (peak 3); humic-like C (peak 4). 

**Figure 3:**
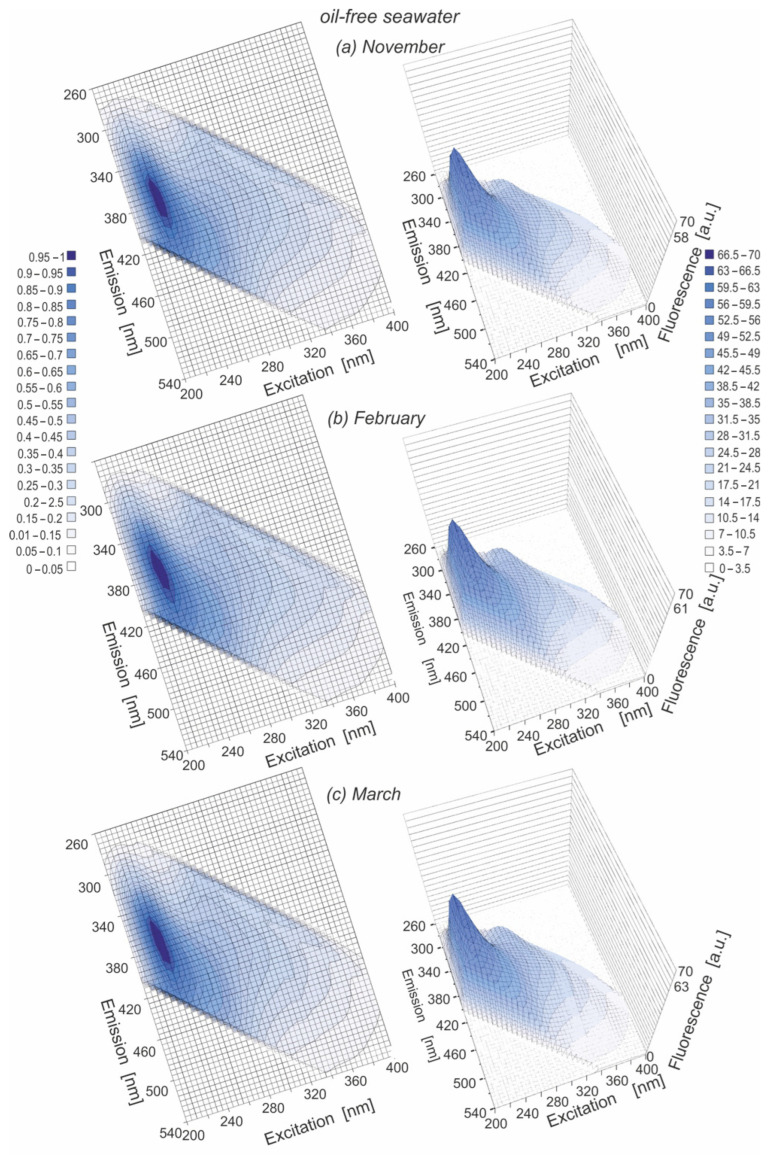
EEMs of oil-free seawater in 2D (**left** side) and 3D (**right** side) for various months: November (**a**), February (**b**) and March (**c**).

Figure 3a–c presents EEMs for oil-free seawater samples using a 2D map (left side of Figure 3) and a 3D map (right side Figure 3) for different months in the period of November 2019 to March 2020. For each month, the main peak 1 (T) was detected, although a shift towards longer emission wavelengths to 370 nm for November and 380 nm for February can be observed. The changes in the wavelength-independent fluorescence maximum (λ_Ex_/λ_Em_) for the main peak T for particular months are presented in Table 4. Moreover, the wavelength-independent fluorescence maximum (λ_Ex_/λ_Em_) was determined for all determined EEM peaks for particular months and is presented in Table 5. To consider the changes in particular peaks in the EEM spectrum for different months from November to March, the fluorescence intensity for particular peaks detected in the EEM spectrum for each month was determined. Table 6 presents the fluorescence intensity of detected peaks linked to their seawater components (T, A, M, C) for particular months (November–March) in the winter season in the Baltic Sea. The fluorescence intensity had low values for determining the particular components in natural seawater in November and December, whereas from January to March, the fluorescence intensity had higher values. The variations in the fluorescence intensity and the positions of the particular peaks in determined EEMs are the confirmation of the changes in CDOM, which can affect light penetration in the seawater column, and has the same influence on biological activities [28], such as primary production [29] and autochthonous production by plankton [30,31]. 

### 3.2. Characteristics of Seawater Artificially Contaminated 

**Figure 4:**
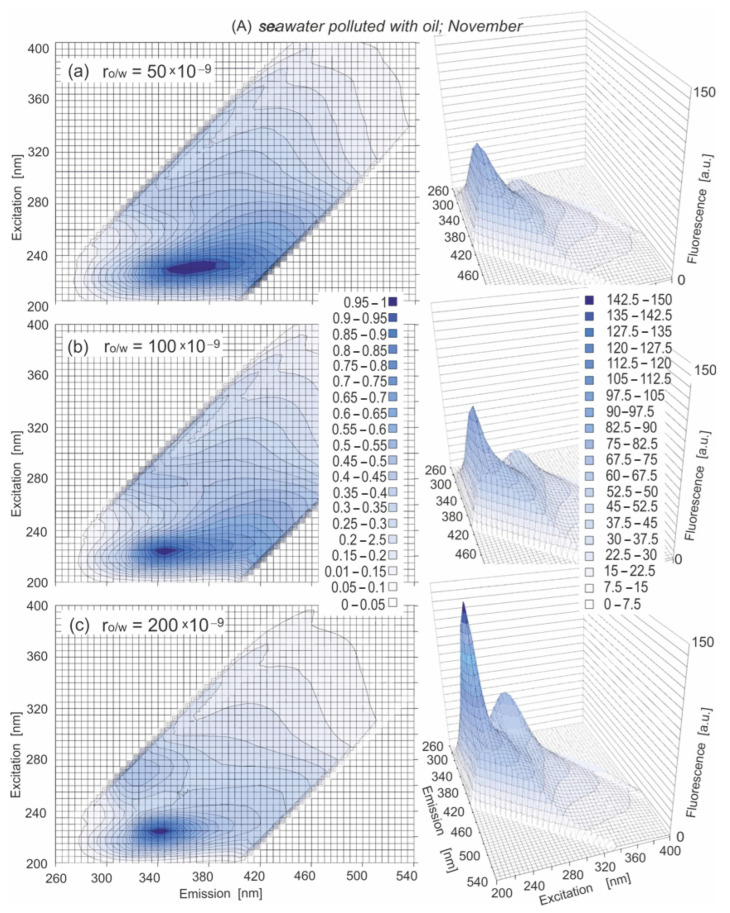

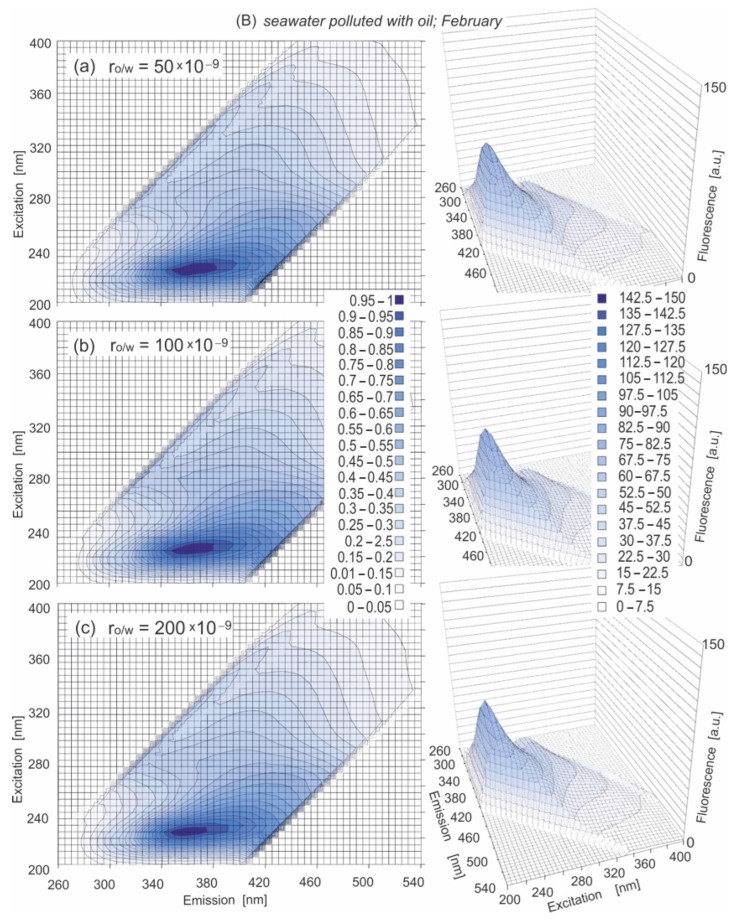

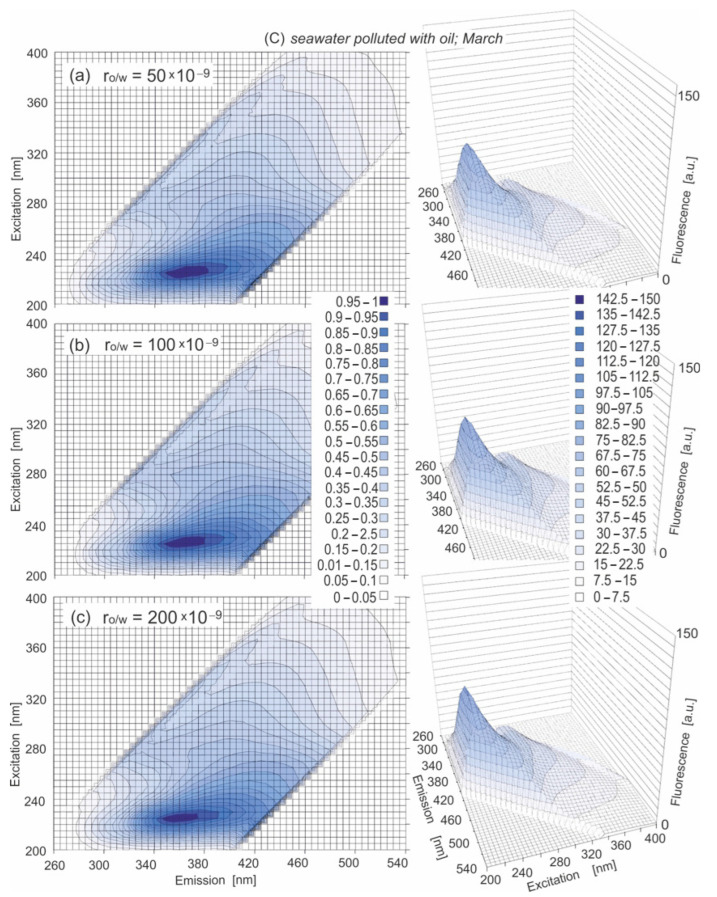
EEMs of seawater polluted by oil in 2D (left side) and 3D (right side) for various r_o/w_: 50 × 10^−9^ (**a**), 100 × 10^−9^ (**b**) and 200 × 10^−9^ (**c**), for various months: November (**A**), February (**B**) and March (**C**).

The influences of the oil added to the seawater samples on the changes in the EEMs in the cold season for particular months (November–March in 2019/2020) and for various r_o/w_ were analyzed. Figure 4A–C presents the EEMs in 2D (left) and in 3D (right) of seawater polluted by a mixture of oils for various r_o/w_ in the chosen months (November, February and March). Based on the obtained EEMs, the major fluorescent peaks for seawater polluted by oil were determined: (225/340) and (275/330). However, the shifting of the position of peak (225/340) to the longer emission wavelengths, from 355 to 370 nm, was determined both for lower r_o/w_ (60 × 10^−9^, 50 × 10^−9^) in November and December and for January, February and March. Moreover, peak (275/330) was determined only for the highest r_o/w_ in November, December and January. The major fluorescent peaks for seawater polluted by oil for each considered month and for all r_o/w_ are presented in Table 7.

When the EEMs of seawater polluted by oil are presented in 3D (right side of Figure 4A–C, the changes in the fluorescence intensity of the detected peaks are visible. The main peak (225/340) achieved higher fluorescence intensity than peak (275/330). For November (Figure 4A), the fluorescence intensity of the main peak depends on the r_o/w_ and increases when the r_o/w_ increases. In February and March, the fluorescence intensity of the main peak is low, which means that the peak was practically undetectable. Moreover, the fluorescence intensity has higher values for November than for February and March. This was probably caused by the influence of the increasing presence of natural seawater constituents, which achieved higher values of fluorescence intensity in March (see Table 1 and Table 6). When the fluorescence intensity of oil-free seawater in comparison to polluted one is considered (Figure 2 and Figure 3), for lower r_o/w_ = 80 × 10^−9^ and 50 × 10^−9^, the changes in fluorescence intensity are minimal. This means that EEMs of oil-free seawater are similar to the EEMs of polluted by oil seawater, and oil detection is impossible.

**Figure 5:**
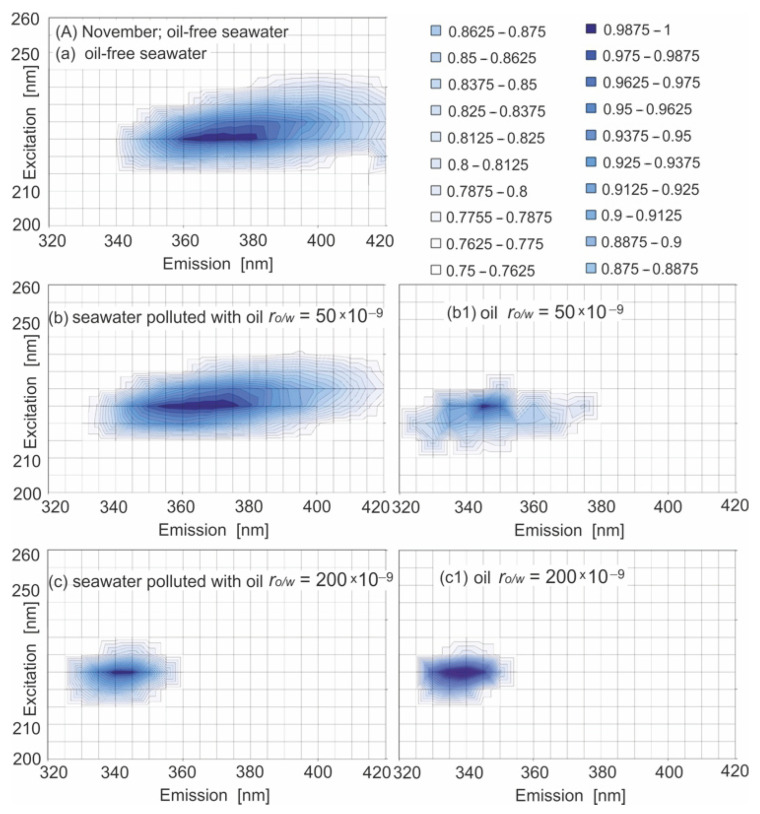

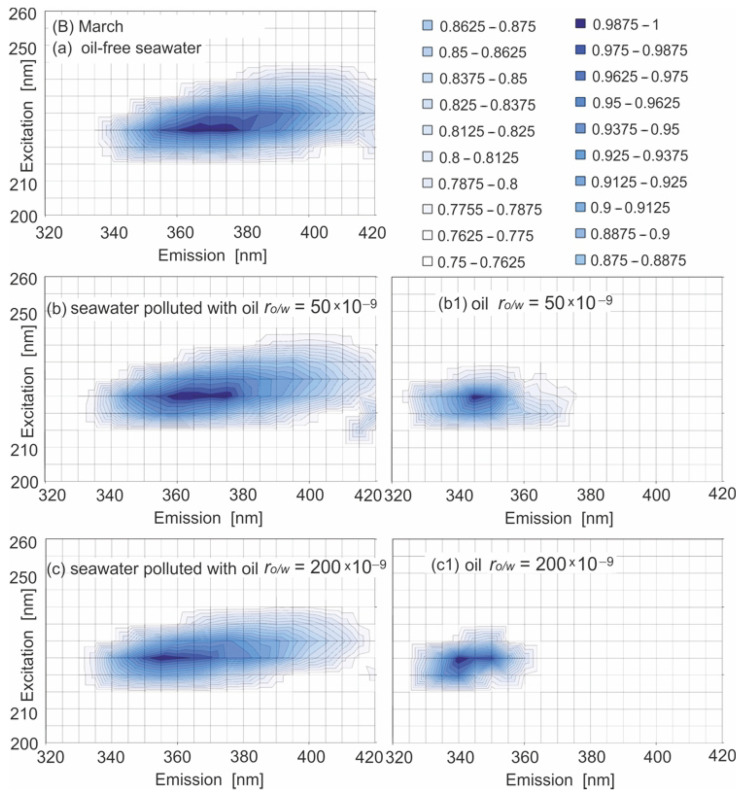
EEMs of oil-free seawater (**a**) and seawater polluted by oil for various r_o/w_: 50 × 10^−9^ (**b**) and 200 × 10^−9^ (**c**) and after the removal of the fluorescence of natural components for various r_o/w_: 50 × 10^−9^ (**b1**) and 200 × 10^−9^ (**c1**), for November (**A**) and March (**B**).

To confirm the presence of oil in seawater for lower r_o/w_, we have to focus on the shifting of the peak positions in relation to oil added to the seawater. Therefore, the emission and excitation wavelengths in the 2D EEM spectrum were limited to 215–245 nm and 320–420 nm, respectively. The EEMs of seawater polluted by oil and oil-free seawater in a limited wavelength range for November and March are presented in Figure 5A,B. The position of peak (225/365) for the seawater polluted by oil for r_o/w_ = 50 × 10^−9^ in relation to oil-free seawater peak (225/370) was shifted minimally to a lower emission wavelengths (about 5 nm) in November (see Figure 5A(b)), whereas for the highest r_o/w_ = 200 × 10^−9^, the significant shafting of the peak to the lower emission wavelength of 340 nm was noticed (see Figure 5A(c)). However, in March (Figure 5B), the EEMs of seawater polluted by oil are similar to those of oil-free seawater, independently of the r_o/w_. In that case, to confirm the presence of oil in seawater, the fluorescence coming from seawater constituents has to be removed. The result of that is presented in Figure 5(A–B(b1–c1)). In that case, peak 225/340 for the highest r_o/w_ and peak 225/345 for the lowest r_o/w_ were determined. The above-mentioned peaks were determined for all considered r_o/w_ in the range from 200 × 10^−9^ to 50 × 10^−9^ and for all considered months. Major fluorescent peaks for oil-polluted seawater for all r_o/w_ with their wavelength-independent fluorescence maxima (λ_Ex_/λ_Em_) are presented in Table 8.

## 4. Discussion

Fluorometric index (*FI*_o/w_) was defined for oil detection in seawater in a quick and easy way, taking into account EEMs of natural seawater and oil-polluted seawater [22,23]. The analysis of the EEMs allowed us to define *FI*_o/w_ while taking into account the fluorescence intensity at the emission wavelength for oil and the intensity of the emission wavelengths for natural (oil-free) seawater corresponding to the determined excitation maxima for both natural seawater and seawater polluted by oil (Formula (1)) [22,23].

(1) $$FI_{o/w}={\left[\frac{I(\lambda_{Emission\;\;of\;seawater\;polluted\;by\;oil})}{I(\lambda_{Emission\;\;of\;natural(oil-free)\;seawater})}\right]}_{\lambda_{Excitation}}$$

The values of emission wavelength for seawater contaminated by oil at 340 nm and for natural (oil-free) seawater at 355 nm, corresponding to the excitation wavelength at 225 nm, were selected based on the determined EEMs (Formula (2)).

(2) $$FI_{o/w}={\left[\frac{I\left(\lambda_{Em=340}\right)}{I\left(\lambda_{Em=355}\right)}\right]}_{\lambda_{Ex=225}}$$

In this study, *FI*_o/w_ was calculated to check its effectiveness in oil detection in the cold season in the r_o/w_ range 50–200 × 10^−9^ for coastal water in the Gulf of Gdansk. Therefore, *FI*_o/w_ was calculated by taking into account the EEMs of oil-free seawater and seawater contaminated by a mixture of oils for different r_o/w_ in November, December, January, February and March based on Formula 2. Table 9 presents determined *FI_o/w_* for oil-free seawater for all considered months. *FI_o/w_* for oil-free seawater ranged from 0.80 to 0.84. Variations in *FI_o/w_* values for the considered months depended on the fluorescence intensity, which changed due to the changing amounts of natural seawater constituents. The results of *FI_o/w_* calculations for seawater polluted with oil for all considered months are presented in Table 10. The obtained *FI_o/w_* values for polluted seawater ranged from 0.90 to 1.51. However, values below 1 were determined three times (0.95, 0.96 and 0.90) and only for low r_o/w_. Moreover, the values were still higher than *FI_o/w_* for oil-free seawater. The highest values of *FI_o/w_* were obtained for November, whereas the lowest values were determined for February and March (which could have been caused by the presence of natural seawater components, such as primary production or phytoplankton (see Table 1)). However, low *FI_o/w_* values in February cannot reflect primary production, which had low values in this month (see Table 1). The idea of oil detection based on *FI*_o/w_ is the signaling of the presence of oil in seawater for *FI*_o/w_ values above 1. For the low r_o/w_ of 50–80 × 10^−9^ for February and March, *FI_o/w_* was 1, and in that case, the oil detection in seawater could be difficult. For this reason, the dependence of the main peak for oil-free seawater on the particular months was considered (see Table 5). For February, peak 225/380 was shifted to a longer emission wavelength of 380 nm, than for other months. This meant that for polluted seawater (Table 6), peak 225/365–375 was detected and was shifted to longer emission wavelengths than for other months. The data for the wavelength-independent fluorescent maxima (λ_Ex_/λ_Em_) for seawater polluted by oil in Table 8 indicate that for February, peaks 225/445 and 225/435 were determined. However, for other months, peak 225/440 was determined. Therefore, when the natural seawater peak is shifted to longer emission wavelengths (in this case, 380 nm), oil detection based on proposed *FI_o/w_* could be disturbed. 

**Figure 6:**
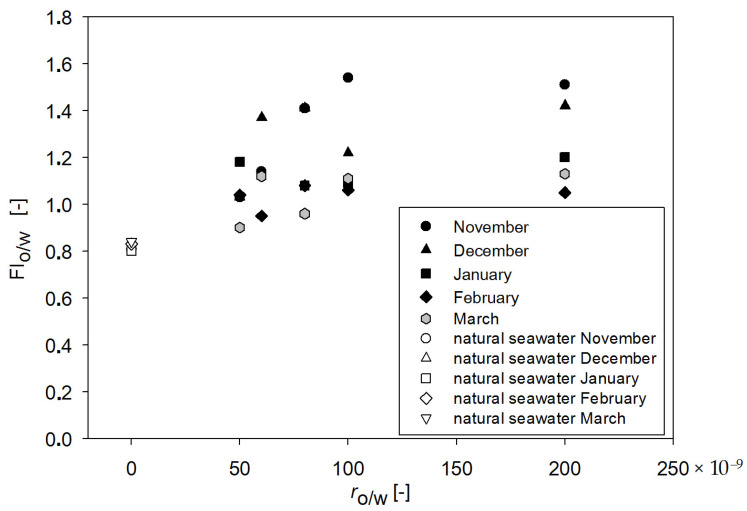
*FI*_o/w_ values for natural seawater and seawater contaminated by a mixture of oil as a function of r_o/w_ for November, December, January, February and March in 2019/2020.

The proposed *FI*_o/w_ could be used in sensors for oil detection. In that case, the dependence on r_o/w_ and time of sampling should be considered. Therefore, the variations in *FI*_o/w_ values from the r_o/w_ among different dates of sampling are considered in Figure 6. The dependence of *FI*_o/w_ on r_o/w_ could be approximated by a constant function in the r_o/w_ range 80–200 × 10^−9^ for all considered months, or 50–80 × 10^−9^ could be approximated as a linear function. This allows us to conclude on the independence of *FI*_o/w_ from r_o/w_ in the range 80–200 × 10^−9^, and probably for higher values of r_o/w_. 

## 5. Conclusions

The method of oil detection in seawater based on a fluorometric index (FI) which could be applied to an oil sensor design was analyzed. The intention was to check the effectiveness of FI for oil detection in seawater sampled from coastal waters during the cold season (late autumn, winter and early spring) in the Baltic Sea region. The results confirm that FI can be a sensitive tool for signaling the presence of oil in seawater. It was found that FL determined from EEM spectra of seawater polluted with oil is not the same in different months. However, the proposed FI indicates sensitivity for oil detection in the seawater for low r_o/w_ (50 × 10^−9^), both when oil is present on the surface of seawater and when it is at various depths in the sea. There are indications that the noted impact of the seawater sampling date may be caused by the seasonal variability of the concentrations of the natural seawater constituents. For example, low FI values were determined for oil-contaminated seawater when the natural constituents of the seawater were at high levels (confirmed in March). Moreover, the results for the FI index indicate that for proper oil detection (especially for low r_o/w_), the amounts of natural constituents of seawater are of significance. This is due to the fact that low levels of them is not a prerequisite for obtaining high FI values in the event of oil entering seawater. The shares of the individual constituents of CDOM are of particular importance, especially that of the tryptophan-like peak. It has a significant impact on the obtained FI values in relation to the oil-contaminated seawater. This study has shown that when the maximum of the emission wavelengths for the tryptophan-like peak is shifted towards longer emission wavelengths for natural seawater (February), low r_o/w_ FI achieves values below 1 that are similar to the values of oil-free seawater. Despite these inconveniences, in the future, FI should prove to be a good indicator of the presence of oil in the vicinity of a fluorescent sensor immersed in seawater.

During the analysis of the ability to detect oil in the depths of the sea, the temporal variability of the fluorescent properties of the seawater was revealed. The mechanism of these changes is not fully identifiable—a separate study of this phenomenon would be needed, involving increased sampling frequency and possibly the determination of other parameters besides the intensities of the peaks in the EMM spectra. However, in this case it was only about confirming the possibility of oil detection despite the changes taking place in the seawater in the five months of the cold season.

## Figures and Tables

**Table 1:** Parameters of seawater sampled from Gdynia station, located in the coastal waters of the Gulf of Gdansk in the southern Baltic Sea in the winter season 2019–2020 (from November 2019 to March 2020) [24].

	November	December	January	February	March
Temperature [°C]	8.75	7.58	5.25	4.5	4.69
Salinity [PSU]	6.2	6.44	6.33	6.18	6.13
Primary production [mg m^−2^ d^−1^]	0.24	0.08	0.03	0.71	44.1
Phytoplankton [mg m^−3^]	9.1	2.1	0.4	1.6	96.6

**Table 2:** Measurement parameters used for the Hitachi F-7000 FL spectrofluorometer.

excitation wavelength [nm]	200–480
excitation sampling interval [nm]	5
emission wavelength [nm]	260–600
emission sampling interval [nm]	5
scan speed [nm/min]	1200
excitation slit [nm]	10
emission slit [nm]	10
emission sampling interval [V]	400
integration time [s]	0.5

**Table 3:** Major fluorescent components of seawater with their wavelength-independent fluorescence maxima (λ_Ex_/λ_Em_) [18,25,26,27].

Seawater Component	Peak Name	Ex_max_ [nm]/Em_max_ [nm]
tyrosine-like	B	225–237/309–321, 275/305–310
tryptophan-like	T	225/340–390, 275/320–350
UVC-humic-like	C	300–370/380–480, 320–360/420–460
UVA-humic-like	A	247–260/380–500, 260/400–460
marine humic-like	M	290–310/370–410
pigment-like	P	398/660

**Table 4:** The wavelength-independent fluorescence maximum (λ_Ex_/λ_Em_) changes for the main peak T—linked to tryptophan-like seawater components—for particular months (November-March) in the winter season in the Baltic Sea.

	Ex_max_ [nm]/Em_max_ [nm] Peak 1 (T)
November	225/370
December	225/365
January	225/365
February	225/380
March	225/365

**Table 5:** Major fluorescent peaks of natural seawater with their wavelength-independent fluorescence maxima (λ_Ex_/λ_Em_) for particular months in the period of November to March.

Ex_max_ [nm] ± 5 [nm]/Em_max_[nm] ± 5 [nm]				
	Peak 1 (T)	Peak 2 (A)	Peak 3 (M)	Peak 4 (C)
November	225/370	260/430	280/380	320/415
December	225/365	260/430	280/380	320/415
January	225/375	260/430	280/380	320/415
February	225/380	260/430	280/380	320/415
March	225/365	260/430	280/380	320/415

**Table 6:** Fluorescence intensity of detected peaks linked to their seawater components (T, A, M, C) for particular months in the winter season in the Baltic Sea (November–March).

Month		Fluorescence Intensity [a.u.]			
	Peak	T	A	M	C
November		57.75	34.62	23.36	17.46
December		58.04	32.20	23.27	16.24
January		65.61	38.67	27.01	20.02
February		61.20	37.58	25.59	18.51
March		62.74	37.49	26.11	18.51

**Table 7:** Major fluorescence peaks for seawater polluted by a mixture of oils at various r_o/w_ with their wavelength-independent fluorescence maxima (λ_Ex_/λ_Em_) in the period of November to March 2019/2020.

Ex_max_ [nm] ± 5 [nm]/Em_max_[nm] ± 5 [nm]		
November	Peak 1	Peak 2
200 × 10^−9^	225/340	270/325
100 × 10^−9^	225/345	
80 × 10^−9^	225/345	
60 × 10^−9^	225/355	
50 × 10^−9^	225/370	
December		
200 × 10^−9^	225/340	270/325
100 × 10^−9^	225/345	
80 × 10^−9^	225/345	
60 × 10^−9^	225/345	
50 × 10^−9^	225/345	
January		
200 × 10^−9^	225/355	270/325
100 × 10^−9^	225/365	
80 × 10^−9^	225/360	
60 × 10^−9^	225/360	
50 × 10^−9^	225/365	
February		
200 × 10^−9^	225/365	
100 × 10^−9^	225/365	
80 × 10^−9^	225/366	
60 × 10^−9^	225/375	
50 × 10^−9^	225/370	
March		
200 × 10^−9^	225/355	
100 × 10^−9^	225/360	
80 × 10^−9^	225/370	
60 × 10^−9^	225/365	
50 × 10^−9^	225/365	

**Table 8:** Major fluorescent peaks for oil polluted seawater after the fluorescence peaks of natural seawater constituents were removed at various r_o/w_ with their wavelength-independent fluorescent maxima (λ_Ex_/λ_Em_), for November to March in 2019/2020.

Ex_max_ [nm] ± 5 [nm]/Em_max_[nm] ± 5 [nm]		
November	Peak 1	Peak 2
200 × 10^−^^9^	225/340	270/330
100 × 10^−^^9^	225/340	270/330
80 × 10^−^^9^	225/340	270/330
60 × 10^−^^9^	225/340	270/330
50 × 10^−^^9^	225/345	270/330
December		
200 × 10^−^^9^	225/340	275/320
100 × 10^−^^9^	225/345	275/320
80 × 10^−^^9^	225/340	280/330
60 × 10^−^^9^	225/340	280/330
50 × 10^−^^9^	225/340	280/330
January		
200 × 10^−^^9^	225/340	
100 × 10^−^^9^	225/340	
80 × 10^−^^9^	225/345	
60 × 10^−^^9^	225/340	
50 × 10^−^^9^	225/340	
February		
200 × 10^−^^9^	225/345	
100 × 10^−^^9^	225/345	
80 × 10^−^^9^	225/335	
60 × 10^−^^9^	220/345	
50 × 10^−^^9^	225/345	
March		
200 × 10^−9^	225/340	
100 × 10^−9^	225/345	
80 × 10^−9^	225/350	
60 × 10^−9^	220/ 340	
50 × 10^−9^	220/ 340	

**Table 9:** *FI*_o/w_ for natural (oil-free) seawater sampled in November, December, January, February and March in 2019/2020.

			FI_o/w_ [-]		
r_o/w_	November	December	January	February	March
natural seawater	0.80	0.82	0.80	0.83	0.84

**Table 10:** *FI*_o/w_ for seawater polluted by a mixture of oils for various r_o/w_ for November, December, January, February and March in 2019/2020.

			*FI*_o/w_ [-]		
r_o/w_	November	December	January	February	March
200 × 10^−9^	1.51	1.42	1.20	1.05	1.13
100 × 10^−9^	1.54	1.22	1.09	1.06	1.11
80 × 10^−9^	1.41	1.41	1.08	1.08	0.96
60 × 10^−9^	1.14	1.37	1.13	0.95	1.12
50 × 10^−9^	1.03	1.03	1.18	1.04	0.90

## Data Availability

Not applicable.

## References

[1] IMO The International Convention for the Prevention of Pollution from Ships (MARPOL), 1973 as Modified by the Protocol of 1978. http://www.imo.org/en/About/conventions/listofconventions/pages/international-convention-for-the-prevention-of-pollution-from-ships-(marpol).aspx.

[2] Gennaro M. (2004). Oil Pollution Liability and Control under International Maritime Law: Market Incentives as an Alternative to Government Regulation. Vand. J. Transnat’l. Law.

[3] Fingas M., Brown C.E., Orcutt J. (2013). Oil spill remote sensing. Earth System Monitoring: Selected Entries from the Encyclopedia of Sustainability Science and Technology.

[4] Alves T.M., Kokinou E., Zodiatis G., Radhakrishnan H., Panagiotakis C., Lardner R. (2016). Multidisciplinary oil spill modeling to protect coastal communities and the environment of the Eastern Mediterranean Sea. Sci. Rep..

[5] Vikas M., Dwarakish G.S. (2015). International conference on water resources, coastal and ocean engineering (ICWRCOE 2015) Coastal Pollution: A Review. Aquat. Procedia.

[6] Fingas M., Brown C.E. (2017). A Review of Oil Spill Remote Sensing. Sensors.

[7] Fingas M. (2019). Marine Oil Spills 2018. J. Mar. Sci. Eng..

[8] Loh A., Ha S.Y., Kim D., Lee J., Baek K., Yim U.H. (2021). Development of a portable oil type classifier using laser-induced fluorescence spectrometer coupled with chemometrics. J. Hazard. Mater..

[9] Leifer I., Lehr W.J., Simecek-Beatty D., Bradley E., Clark R., Dennison P., Hu Y., Matheson S., Jones C.E., Holt B. (2012). State of the art satellite and airborne marine oil spill remote sensing: Application to the BP Deepwater Horizon oil spill. Remote Sens. Environ..

[10] Jha M.N., Levy J., Gao Y. (2008). Advances in Remote Sensing for Oil Spill Disaster Management: State-of-the-Art Sensors Technology for Oil Spill Surveillance. Sensors.

[11] Zielinski O., Hengstermann T., Robbe N. (2006). Detection of oil spills by airborne sensors. Marine Surface Films.

[12] Fingas M. (2016). Oil Spill Science and Technology.

[13] Viallefont-Robinet F., Roupioz L., Caillault K., Foucher P. (2021). Remote sensing of marine oil slicks with hyperspectral camera and an extended database. J. Appl. Remote Sens..

[14] Conmy R.N., Coble P.G., Farr J., Wood A.M., Lee K., Pegau W.S., Walsh I.D., Koch C.R., Abercrombie M.I., Miles M.S. (2014). Submersible Optical Sensors Exposed to Chemically Dispersed Crude Oil: Wave Tank Simulations for Improved Oil Spill Monitoring. Environ. Sci. Technol..

[15] Hou Y., Li Y., Liu B., Liu Y., Wang T. (2017). Design and Implementation of a Coastal-Mounted Sensor for Oil Film Detection on Seawater. Sensors.

[16] Ferdinand O.D., Friedrichs A., Miranda M.L., Voß D., Zielinski O. (2017). Next-generation fluorescence sensor with multiple excitation and emission wavelengths—NeXOS MatrixFlu-UV. Proceedings of the Oceans-2017.

[17] Shi Y., Xu Y., Jiang F., Sun Z., Wang G., Zeng Z., Gao C., Xue Q., Xue L. (2021). On-site marine oil spillage monitoring probes formed by fixing oxygen sensors into hydrophobic/oleophilic porous materials for early-stage spottypollution warning. RSC Adv..

[18] Baszanowska E., Otremba Z. (2015). Modification of optical properties of seawater exposed to oil contaminants based on excitation-emission spectra. J. Eur. Opt. Soc. Rapid Publ..

[19] Baszanowska E., Otremba Z. (2017). Fluorometric index for sensing oil in the sea environment. Sensors.

[20] Baszanowska E., Otremba Z. (2020). Seawater fluorescence near oil occurrence. Sustainability.

[21] Baszanowska E., Otremba Z. (2020). Synchronous fluorescence spectra of water contaminated by dispersed crude oil. Opt. Appl..

[22] Baszanowska E., Otremba Z. (2019). Detecting the Presence of Different Types of Oil in Seawater Using a Fluorometric Index. Sensors.

[23] Baszanowska E., Otremba Z. (2022). Fluorometric Detection of Oil Traces in a Sea Water Column. Sensors.

[24] Ecohydrodynamic Forecast for the Baltic Sea. http://model.ocean.univ.gda.pl/php/frame.php?area=ZatokaGdanska.

[25] Coble P.G. (1996). Characterization of marine and terrestrial DOM in seawater using excitation-emission matrix spectroscope. Mar. Chem..

[26] Coble P. (2013). Colored dissolved organic matter in seawater. Subsea Optics and Imaging.

[27] Drozdowska V., Freda W., Baszanowska E., Rudź K., Darecki M., Heldt J., Toczek H. (2013). Spectral properties of natural and oil-polluted Baltic seawater—results of measurements and modelling. Eur. Phys. J. Spec. Top..

[28] Mopper K., Kieber D.J., Hansell D.A., Carlson C.A. (2002). Photochemistry and the cycling of carbon, sulfur, nitrogen and phosphorus. Biogeochemistry of Marine Dissolved Organic Matter.

[29] Kowalczuk P., Durako M.J., Young H., Kahn A.E., Cooper W.J., Gonsior M. (2009). Characterization of dissolved organic matter fluorescence in the South Atlantic Bight with use of PARAFAC model: Interannual variability. Mar. Chem..

[30] Kowalczuk P., Stedmon C.A., Markager M. (2006). Modeling absorption by CDOM in the Baltic Sea from season, salinity and chlorophyll. Mar. Chem..

[31] Whitehead R.F., de Mora S., Demers S., Gosselin M., Monfort P., Mostajir B. (2000). Interactions of ultraviolet-B radiation, mixing, and biological activity on photobleaching of natural chromophoric dissolved organic matter: A mesocosm study. Limnol. Oceanogr..

